# Imaging surveillance for the detection of ipsilateral local tumor recurrence in patients who underwent oncoplastic breast-conserving surgery with acellular dermal matrix: abbreviated MRI versus conventional mammography and ultrasonography

**DOI:** 10.1186/s12957-021-02403-2

**Published:** 2021-09-27

**Authors:** Mi Young Kim, Young Jin Suh, Yeong Yi An

**Affiliations:** 1 Department of Radiology, Konkuk University Medical Center, Konkuk University School of Medicine, Seoul, Republic of Korea; 2 Department of Surgery, Division of Breast and Thyroid Surgical Oncology, St. Vincent’s Hospital, College of Medicine, The Catholic University of Korea, Suwon, South Korea; 3 Department of Radiology, St. Vincent’s Hospital, College of Medicine, The Catholic University of Korea, Suwon, South Korea

**Keywords:** Breast cancer, Recurrence, Surveillance, Magnetic resonance imaging, Breast-conserving surgery, Acellular dermal matrix

## Abstract

**Background:**

To evaluate the usefulness of surveillance-abbreviated magnetic resonance imaging (AB-MRI) for the detection of ipsilateral local tumor recurrence (LTR) in patients who underwent oncoplastic breast-conserving surgery (BCS) with acellular dermal matrix (ADM) by comparing its diagnostic performance with that of mammography (MG) and ultrasonography (US).

**Methods:**

The postoperative MG, US, and AB-MRI findings of the ipsilateral breast and pathological results of 324 patients who underwent oncoplastic BCS using ADM were reviewed. The cancer detection rate (CDR), sensitivity, specificity, positive predictive value (PPV) for biopsy, accuracy, and area under the curve (AUC) for each imaging modality were calculated.

**Results:**

A total of 8 ipsilateral LTRs were diagnosed, and most cancers (87.5%) were stage 0 or 1 (median size of invasive cancer, 1.44 cm; range, 0.7–2 cm). The CDRs of MG, US, MG + US, and AB-MRI were 0.009, 0.012, 0.015, and 0.025 per woman, respectively. Three cancers were detectable on only AB-MRI, and the additional CDR of AB-MRI was 0.010. The sensitivity and specificity of MG, US, MG + US, and AB-MRI were 37.5%, 50%, 62.5%, and 100% and 99.7%, 98.4%, 98.1%, and 97.8%, respectively. The PPVs for biopsy were 75%, 44.4%, 45.5%, and 53.3%, respectively. The AUC was significantly higher for AB-MRI (0.989) than for MG alone (0.686, *P* = 0.0009), US alone (0.742, *P* = 0.009), and MG + US (0.803, *P* = 0.04). A total of 66.7% of cancers visible on only AB-MRI were located at the deep posterior portion of the excision cavity, which might have been missed with MG or MG + US due to masking by the ADM filler.

**Conclusion:**

AB-MRI can improve the detection of ipsilateral LTR despite postoperative changes caused by ADM fillers compared to conventional MG and US. Patients who undergo BCS with ADM can be candidates for AB-MRI surveillance considering improved cancer detection and high sensitivity.

## Background

Breast-conserving surgery (BCS) combined with postoperative radiotherapy has been established as a standard treatment for early-stage breast cancer [1]. The goal of BCS is to remove breast cancer completely with an adequate resection margin and without compromising cosmetic outcomes [2, 3]. Achieving both goals may be challenging, and approximately 30% of patients who undergo BCS are known to be unsatisfied with their cosmetic outcome [4, 5]. In particular, large-volume resection during BCS can result in unsatisfactory cosmetic outcomes in women with small- to medium-sized breasts [6–9]. In recent years, oncoplastic surgery has been introduced to overcome the cosmetic disadvantage of conventional BCS and has increasingly gained acceptance among both surgeons and patients [2]. Oncoplastic surgery is a combination of tumor removal and breast reconstruction and is divided into two broad techniques: volume displacement and volume replacement. In patients with relatively small breasts, the volume replacement technique has shown better cosmetic results than the volume displacement technique [6–9].

**Fig. 1:**
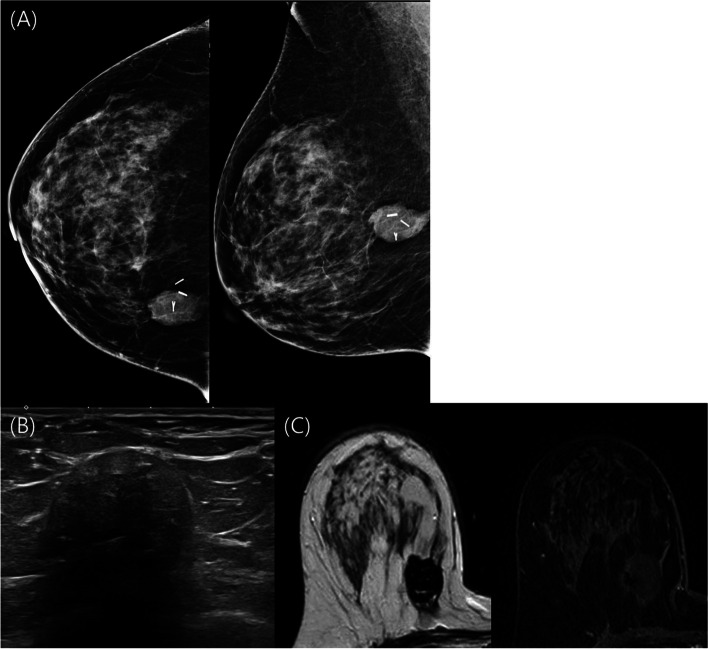
Follow-up imaging after 24 months of a 49-year-old woman who underwent oncoplastic BCS with ADM. The ADM was observed as **A** a circumscribed high-density mass on mammography and **B** a heterogeneous echogenic mass with posterior acoustic shadowing. The evaluation of the deep posterior margin of the excision cavity was limited by shadowing. The ADM showed low signal intensity on T2-weighted imaging and no enhancement on contrast-enhanced T1-weighted imaging (**C**)

Acellular dermal matrices (ADMs) are well-known biological scaffolds of human, bovine, or porcine origin and do not evoke an immune response. ADMs are widely used in burn care and breast reconstruction surgery [10–12]. In breast surgery, ADM is used in more than 75% of immediate tissue expander reconstruction procedures to support the implant [13]. ADMs are increasingly utilized in BCS for volume replacement, and recent reports show satisfactory results regarding their safety and cosmetic outcome over a short-term follow-up period [14–16]. However, concern still exists regarding the influence of postoperative changes by ADM on monitoring local tumor recurrence (LTR) on postoperative imaging surveillance [16, 17]. The ADM-filled cavity presents as a high-density mass-like lesion on mammography (MG) and an echogenic mass with posterior shadowing on ultrasonography (US) [14, 16, 17] (Fig. 1). ADM can cause difficulties in image interpretation using conventional imaging, such as MG and US, during the follow-up period [14, 16, 17]. To date, whether ADM inserted in the excision cavity interferes with the early diagnosis of ipsilateral LTR has not yet been reported.

Breast magnetic resonance imaging (MRI) is the most sensitive imaging method for detecting breast cancer and offers the highest cancer detection rate (CDR) of all breast imaging modalities. Since the introduction of the abbreviated MRI (AB-MRI) protocol by Kuhl et al. [18, 19], it has competed with MG or US as a screening tool by reducing the complexity and cost of breast MRI with a short scan time and improving access to breast MRI [20]. Currently, the use of AB-MRI as a screening tool is being actively investigated at different risk levels. We hypothesize that AB-MRI can improve the detection of ipsilateral LTR despite postoperative findings with ADM insertion compared to conventional MG or US. Therefore, the purpose of this study is to compare the diagnostic performance of AB-MRI to that of MG and US for the detection of ipsilateral LTR and to evaluate the clinical value of AB-MRI in the postoperative surveillance of patients who underwent oncoplastic BCS with ADM.

## Materials and methods

### Study population

This retrospective study was approved by the Catholic Medical Center Office of the Human Research Protection Program (CMC-OHRP) Institutional Review Board (Approval No. VC21RISI0105), and the requirement for informed consent was waived. In a retrospective search of our medical database between August 2017 and June 2020, we identified 329 patients with stage 0 to III breast cancer who underwent BCS and immediate volume replacement with the crosslinked human ADM (MegaDerm; L&C BIO, Seongnam, Korea) derived from donated human skin in the USA tissue banks following the guidelines of the American Association of Tissue Banks and the US Food and Drug Administration. Our study included patients who underwent postoperative imaging surveillance using MG, US, and AB-MRI and were followed-up for at least 1 year. Five patients were excluded due to follow-up loss within 1 year (*n* = 5). A total of 324 patients with a median follow-up of 22.8 months (12–38 months) were included in this study. The patients ranged in age from 22 to 83 years, and the mean age was 53.9 years. We reviewed the medical records, including the clinical, radiological, and pathological characteristics of breast cancer of the study population.

### Definition

An ipsilateral LTR was defined as a recurrent invasive carcinoma or ductal carcinoma in situ (DCIS) that occurred in either the parenchyma and/or skin of the treated breast. An ipsilateral LTR was confirmed by histopathologic evaluation of the tumor. The interval was calculated from the date of the first oncoplastic BCS procedure with ADM to the date of pathologic confirmation.

### Postoperative imaging surveillance

After breast cancer surgery, all patients underwent follow-up examinations with MG and US every 6 months for the first 2 years and annually thereafter. All mammographic imaging data were acquired by using a full-field digital mammography (DM) unit with integrated digital breast tomosynthesis (DBT) acquisition (Selenia Dimensions, Hologic). Standard DM followed by DBT acquisition was performed during the same breast compression and included two bilateral view mammograms (craniocaudal and mediolateral oblique views). Whole-breast US examinations were obtained by using an Aplio i800 system (Canon Medical System) equipped with a matrix linear transducer with a bandwidth of 5 to 18 MHz. With a 3 T scanner (Verio, Siemens Medical Solutions, Erlangen, Germany) equipped with a dedicated breast coil, AB-MRI was performed by using the following protocols: (a) axial fat-suppressed T2-weighted imaging, (b) pre- and postcontrast axial T1-weighted imaging before and immediately after gadoterate meglumine injection (0.1 mmol per kilogram body weight, Dotarem; Guerbet, Anlnay-Sous-Bois, France), (c) subtraction from postcontrast T1-weighted imaging, and (d) reformatting with a maximum intensity projection. The total acquisition time was only 8.3 min. AB-MRI was performed along with MG and US examinations on the same day or around the same time.

### Postoperative imaging interpretation

Two board-certified radiologists specializing in breast imaging with 17 years of experience who were blinded to the results of the other studies independently reviewed MG, US, and AB-MRI follow-up studies. Because we aimed to evaluate the influence of ADM on ipsilateral LTR, we analyzed imaging studies in the treated breast only. All imaging studies were interpreted according to the 5th edition of the Breast Imaging Reporting and Data System (BI-RADS) classification. BI-RADS category 4 or 5 was considered a positive result, and tissue diagnosis was recommended. For lesions categorized as BI-RADS category 3, short-interval follow-up (6–12 months) was recommended. If lesions were stable during the follow-up period, they were downgraded to BI-RADS category 2. If any changes occurred, the lesions were upgraded to BI-RADS category 4, and biopsy was recommended. If a suspicious lesion was visible on MG or US, MG-guided or US-guided biopsy was performed. If a suspicious lesion was detected on only AB-MRI, a second-look US was first performed. If a correlate was present on second-look US, US-guided biopsy was performed. If there was no correlation, MR-guided biopsy was recommended, but none of the patients in this study were diagnosed by this modality.

### Outcome measures and statistical analysis

We used the tissue diagnosis results at biopsy and at the clinical follow-up within 1 year as the reference standard to assess imaging surveillance for the detection of ipsilateral LTR. A true positive was defined as a case with positive results on MG, US, the combination of MG and US (MG + US), and AB-MRI followed by tissue confirmation. A true negative was defined as a case with negative imaging findings and the absence of cancer at the 1-year surveillance imaging. A false positive was defined as a case with positive imaging findings and with no detection of cancer within 1 year. A false negative was defined as a case with negative imaging findings and a tissue confirmation of cancer within 1 year.

We calculated the CDR, positive predictive value (PPV) for biopsy, sensitivity, specificity, diagnostic accuracy, and area under the curve (AUC) of MG, US, MG + US, and AB-MRI surveillance. Additionally, we compared the diagnostic performance outcomes of MG, MG + US, and AB-MRI surveillance by using receiver operating characteristic (ROC) analysis. The CDR was defined as the number of detected malignancies per woman for each group.

The clinicopathological characteristics of primary breast cancer patients with and without ipsilateral LTR were compared using the independent samples *t* test or Fisher’s exact test for continuous and categorical variables. Statistical analyses were performed using SAS version 9.4 (SAS Institute, Cary, NC, USA) and MedCalc ver. 16.1 (MedCalc software, Mariakerke, Belgium). Statistical significance was set at *P* value < 0.05.

## Results

The demographic details of the patients in this study and the characteristics of the patients with ipsilateral LTR detected on imaging surveillance are summarized in Table 1. A total of 8 ipsilateral LTRs were diagnosed, and the frequency of LTR was 2.5% (8 of 324 women) in this study. Tumor subtype was significantly different between patients with and without ipsilateral LTR. HER2 (+) breast cancer and triple-negative breast cancer, not luminal-type breast cancer, were significantly associated with ipsilateral LTR (*P* = 0.03). The other findings were not significantly different.

**Table 1:** Characteristics of 324 patients included in this study

	No ipsilateral LTR (*n* = 316)	Ipsilateral LTR (*n* = 8)	*P* value
Age			0.52
< 50 years	114	2	
≥ 50 years	202	6	
Histopathology			0.38
DCIS	51	0	
IDC	227	8	
ILC	9	0	
Others	29	0	
TNM stage			0.57
Stage 0	53	0	
Stage I	133	4	
Stage II	108	3	
Stage III	19	1	
Tumor subtype			0.03
LumA/B	241	3	
HER2	36	3	
Triple negative	39	2	
Ki-67			0.50
Low (< 14%)	100	1	
High (≥14%)	216	7	
HG			0.19
Gr1	57	0	
Gr2	119	3	
Gr3	68	4	
N/A	72	1	
NG			0.71
Gr1	27	0	
Gr2	129	3	
Gr3	142	4	
N/A	18	1	
LI			0.61
(+)	63	2	
(−)	196	6	
N/A	57	0	
PNI			0.39
(+)	24	1	
(−)	233	7	
N/A	57	0	
VI			0.39
(+)	3	0	
(−)	256	8	
N/A	57	0	
Nodal status			0.42
(+)	79	3	
(−)	237	5	
NAC			0.10
(+)	50	3	
(−)	266	5	
Adjuvant CTx			0.64
(+)	212	6	
(−)	104	2	

**Fig. 2:**
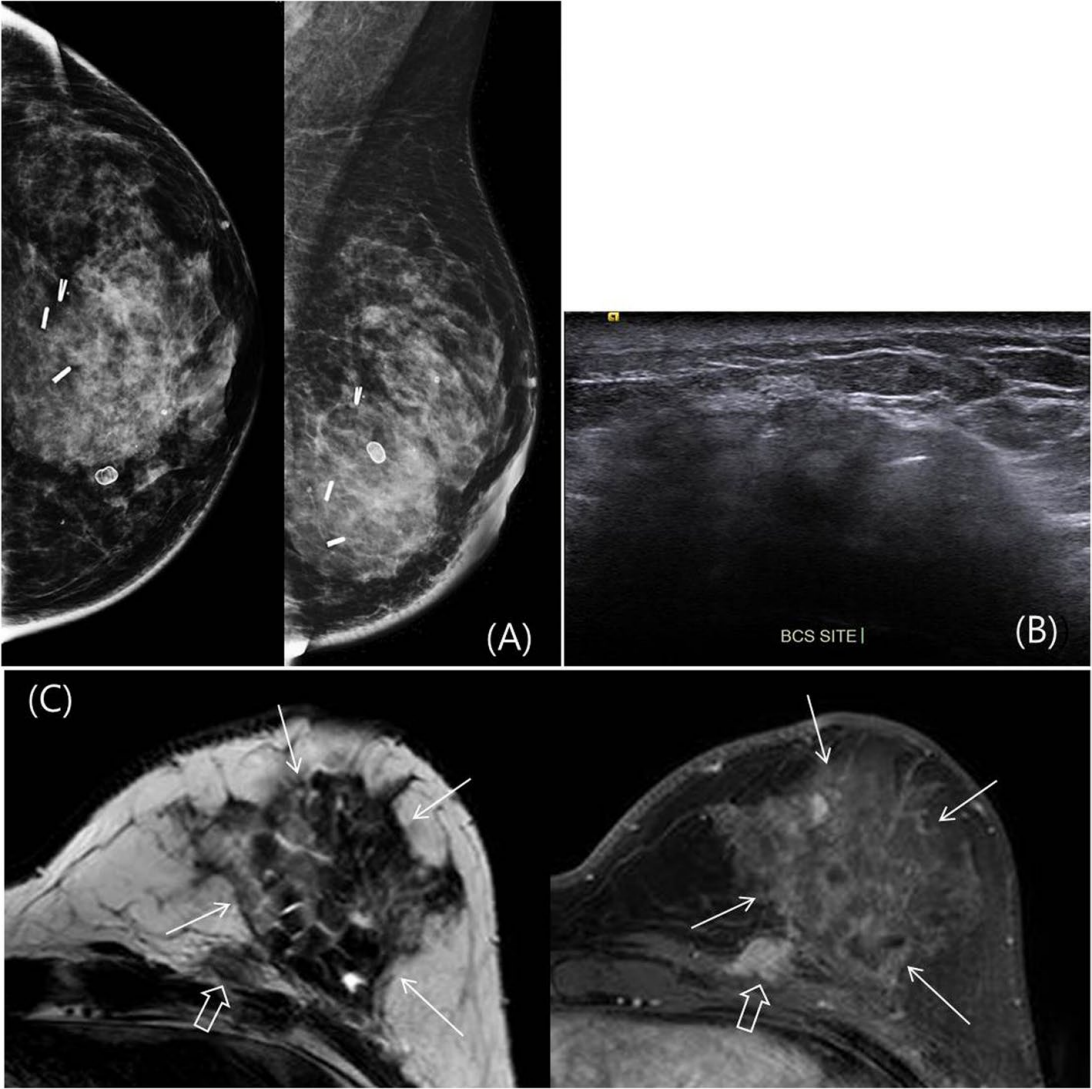
Follow-up imaging after 6 months of a 56-year-old woman who underwent oncoplastic BCS with ADM. **A**, **B** Post-surveillance mammogram and ultrasonogram were negative. **C** AB-MRI showed an irregular enhancing mass (block arrow) at the deep margin of the excision cavity filled with ADM (arrows). Second-look ultrasonography was performed, but the lesion at the deep portion was not identified due to shadowing. At surgery, a 0.7-cm-sized invasive cancer was confirmed

The types and biological profiles of ipsilateral LTR are summarized in Table 2. Of 324 patients, 15 patients had suspicious malignant lesions categorized as BI-RADS 4 or 5, and biopsies were performed. The biopsy recommendation rates of MG, US, and AB-MRI were 1.2% (4 of 324), 2.8% (9 of 324), and 4.6% (15 of 324), respectively. Of these 15 suspicious lesions, 8 were malignant (6 invasive, 2 ductal carcinoma in situ) and 7 were benign. Most of the detected cancers were stage 0 or 1 (87.5%, 7 of 8), and the median size of invasive cancer was 1.44 cm (range 0.7–2 cm). A total of 62.5% (5 of 8) were high-grade tumors, and 25% (2 of 8) showed multifocalities. One patient had clinically detected skin lesions, and invasive cancer was diagnosed by skin punch biopsy. After mastectomy, cancer invading into the dermis/epidermis with skin ulceration (T4b) was confirmed in the specimen. The remaining 7 patients were clinically asymptomatic, and imaging detected cancers. As summarized in Table 2, MG detected 3 cancers, US detected 4 cancers, and AB-MRI detected all 8 cancers. AB-MRI detected 3 additional cancers compared to MG + US. Overall, the CDRs of MG, USG, MG + US, and AB-MRI were 0.009, 0.012, 0.015, and 0.025 per woman, respectively. The additional CDR of AB-MRI was 0.010 per woman. Of the 3 cases visible only on AB-MRI, two cancers were observed at the medio-basal margin and at the basal margin of the original tumor bed (deep posterior portion of ADM filled cavity), so recurrent tumors were partially and completely obscured by ADM filling on both MG and US (Fig. 2) and missed on initial US exam. One lesion was identified on second-look US and confirmed by US-guided biopsy, but another lesion was not identified on second-look US, so ADM removal and surgical excision were performed. One patient had clinically detected skin lesions (reddish patches on skin), which were obscured by radiation treatment-related skin edema on both MG and US. Tumor infiltration presented as overlying skin enhancement and scattered small foci of remaining breast parenchyma (less than 0.3 cm) on AB-MRI.

**Table 2:** Characteristics of 8 ipsilateral LTRs detected on imaging studies

No.	Age (years)	Primary cancer				Detected cancer								
		Pathology	Subtype	Grade	Stage	Interval (mo)	Detection method	MG	US	MRI	Location	Pathology	Size (cm)	Subtype
19	56	IDC	HER2	Gr3	I	30	Imaging	+	+	+	Medial	DCIS	5	HER2
121	56	IDC	LumA	Gr2	I	8	Imaging	–	+	+	Lateral	IDC	1.2	LumA
139	43	IDC	HER2	Gr3	I	6	Imaging	–	–	+	Medial, basal	IDC	1.8	HER2
150	58	IDC	TN	Gr3	II	19	Imaging	+	+	+	Medial, lateral	IDC	2	TN
266	56	IDC	HER2	Gr3	II	12	Imaging	–	–	+	Basal	IDC	0.7	HER2
280	48	IDC	LumA	Gr2	II	5	Imaging	–	+	+	Superficial	IDC	1.5	LumA
281	57	IDC	TN	Gr3	III	7	Clinical	–	–	+	Skin	IDC	Skin	TN
313	71	IDC	LumA	Gr2	I	7	Imaging	+	–	+	medial	DCIS	0.5	LumA

**Fig. 3:**
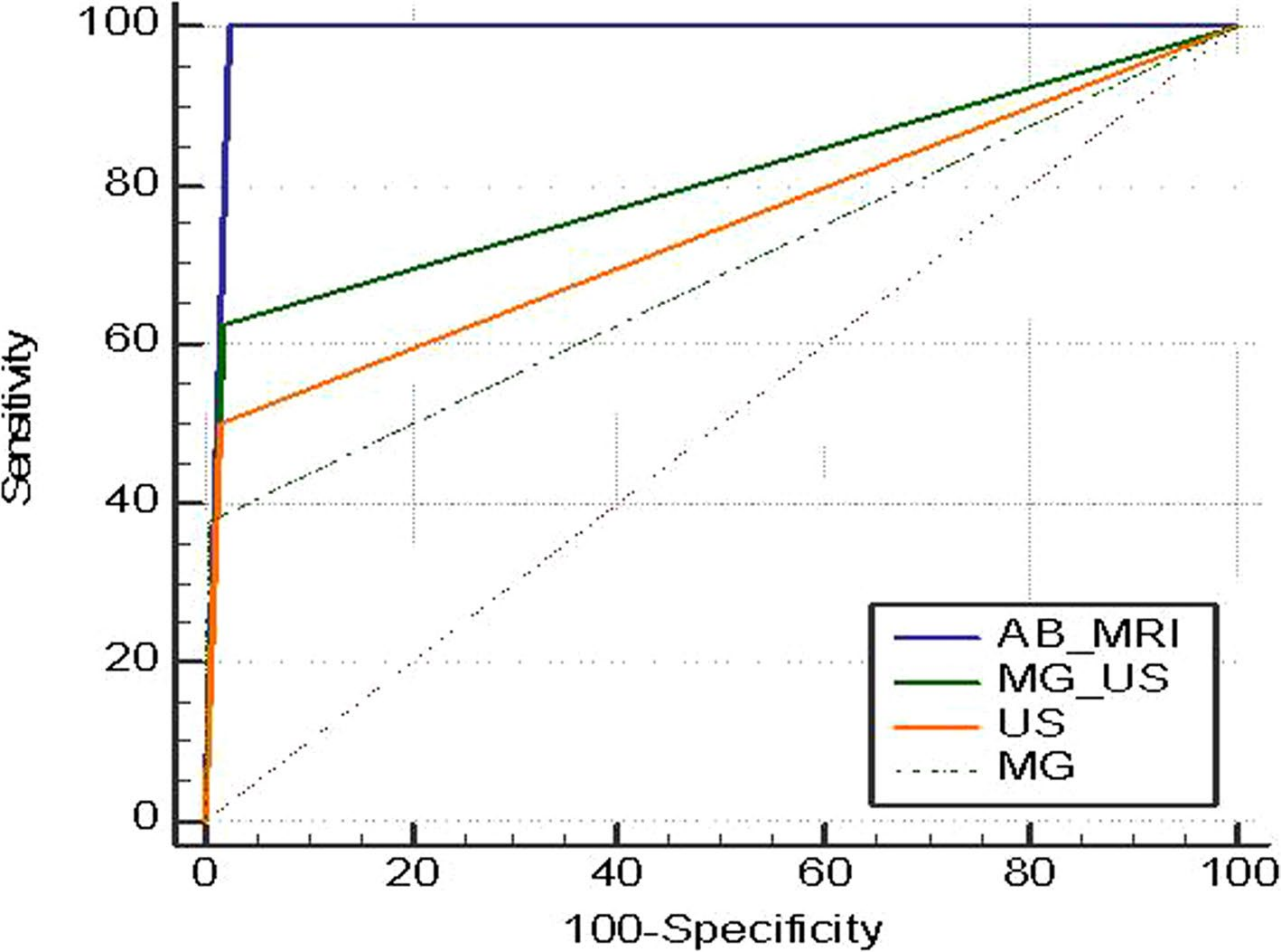
Comparison of ROC curves (AB-MRI ~ MG + US, *P* = 0.04; AB-MRI ~ MG, *P* = 0.0009; AB-MRI ~ US, *P* = 0.009)

A comparison of the diagnostic performance of each imaging modality for the detection of ipsilateral LTR is summarized in Table 3, and the corresponding ROC curve is shown in Fig. 3. The sensitivity and specificity of MG, US, MG + US, and AB-MRI were 37.5%, 50%, 62.5%, and 100% and 99.7%, 98.4%, 98.1%, and 97.8%, respectively. The PPVs for biopsy were 75%, 44.4%, 45.5%, and 53.3%, respectively. The AUC was significantly higher for AB-MRI (0.989, 95% CI 0.971–0.997) than MG alone (0.686, 95% CI 0.632–0.736; *P* = 0.0009), US alone (0.742, 95% CI 0.691–0.789; *P* = 0.009), and MG + US (0.803, 95% CI 0.755–0.845; *P* = 0.04). There were no statistically significant differences in the AUC values between MG and US, MG, and MG + US, or US and MG + US.

**Table 3:** Diagnostic performances of each imaging modalities

	Sensitivity (%)	Specificity (%)	PPV (%)	NPV (%)	Accuracy (%)	AUC
MG	37.5 (8.5–75.5)	99.7 (98.3–100.0)	75.0 (25.9–96.3)	98.4 (97.4–99.1)	98.2 (96.0–99.3)	0.686 (0.632–0.736)
USG	50.0 (15.7–84.3)	98.4 (96.3–99.5)	44.4 (20.8–70.9)	98.7 (97.5–99.4)	97.2 (94.8–98.7)	0.742 (0.691–0.789)
MG + USG	62.5 (24.5–91.5)	98.1 (95.9–99.3)	45.5 (24.2–68.5)	99.0 (97.7–99.6)	97.2 (94.8–99.6)	0.803 (0.755–0.845)
AB-MRI	100.0 (63.1–100.0)	97.8 (95.5–99.1)	53.3 (35.5–70.4)	100 (98.4–100)	97.8 (95.6–99.1)	0.989 (0.971–0.997)

## Discussion

The aim of surveillance in breast cancer survivors is to detect second breast cancers in the asymptomatic phase, which allows interventions that may lead to improved survival and quality of life. Because the early detection of tumor recurrence can result in a better prognosis, careful clinical and imaging surveillance are needed for patients who undergo BCS. Although there is no standard protocol for posttreatment imaging surveillance, annual MG with or without US is currently used in women who underwent BCS [21–23].

In patients who underwent oncoplastic BCS with ADM, ipsilateral LTR is an important issue because it is associated with distant metastasis and poor prognosis and is directly related to remnant tumor cells within the remaining breast tissue. However, there are concerns that the fibrogenetic action induced by ADM and its partial reabsorption may lead to misdiagnosis during follow-up [17]. Biomaterials such as ADM or regenerated oxidized cellulose show similar peculiar imaging findings, which are well-circumscribed masses that are similar in density and echogenicity to fibroglandular tissue on both MG and US [14, 16, 24, 25]. Thus, the early and accurate diagnosis of ipsilateral LTR using only conventional imaging modalities such as MG and US can be problematic in women who undergo oncoplastic BCS with ADM.

MRI is often used as a part of postoperative surveillance in the clinical setting and is considered the most sensitive imaging modality compared with conventional imaging methods such as MG and US in discriminating between postoperative scarring and tumor recurrence [26, 27]. Current data also support MRI as a postoperative surveillance modality with high diagnostic yield, sensitivity, and specificity for detecting recurrent cancer [26, 28–32]. More recently, the American College of Radiology has broadened its stance to also recommend supplemental screening MRI in women with a personal history of breast cancer [33]. The reason that most guidelines do not support the use of breast MRI for postoperative surveillance is its comparative effectiveness [34–37]. However, the use of the AB-MRI protocol with a short examination time is more cost-effective [19]. AB-MRI also showed equivalent diagnostic accuracy compared to full diagnostic MRI protocols [18], which has led to the consideration of using AB-MRI as a screening modality in patients with a personal history of breast cancer.

In our study, the ipsilateral LTR rate was 2.5% in patients who underwent oncoplastic BCS with ADM, which was similar to the previously reported rate in patients who underwent conventional BCS (2%) [38, 39]. Tumor subtype was different between patients with and without LTR. HER2 (+) type cancer and triple-negative type cancer were significantly associated with ipsilateral LTR in univariate analysis, although multivariate analysis was not performed. Regarding the performance of AB-MRI in the detection of LTR, AB-MRI demonstrated a significantly higher invasive CDR than other imaging modalities. AB-MRI detected 15 more cancers per 1000 women than MG alone and 9 more cancers per 1000 women than MG + US. Most detected cancers on AB-MRI were stage 0 or 1 (87.5%, 7 of 8), except for only 1 cancer with skin metastasis (T4b, stage IIIC), which was comparable to previous results indicating that MRI could detect biologically relevant breast cancer in high-risk patients [20, 40–44]. In addition to improved CDR, AB-MRI showed the highest sensitivity and negative predictive value (NPV) of 100% without sacrificing specificity for detecting LTR, and its AUC value was higher than that of other surveillance modalities (0.989 for AB-MRI vs. 0.686 for MG, 0.742 for US, and 0.803 for MG + US), which was consistent with previous data that MRI had a higher cancer detection yield, high sensitivity and specificity, and acceptable PPV for biopsy [28–30, 36, 45–51].

Despite the high sensitivity and CDR of AB-MRI, its high false positive rate (25%) and biopsy recommendation rate (4.6%) are the main drawbacks of its use in surveillance. However, 62.5% of cancers (5 of 8) might have been missed with mammography alone, and 37.5% (3 of 8) might have been missed with MG + US. In particular, 66.7% (2 of 3) of cancers visible on only AB-MRI were located at the basal margin of the excision cavity, which were obscured partially and completely by the ADM filler on conventional imaging. 50% (1 of 2) of cancers at the basal margin of the excision cavity were still not identified on a second-look US exam due to masking by the ADM filler. Therefore, we believe that AB-MRI surveillance could be a useful screening tool for the detection of ipsilateral LTR without delayed diagnosis despite postoperative changes with ADM filler in these patients. If informed patients choose AB-MRI for surveillance considering the harm caused by false negative cases, the false positive cases resulting from AB-MRI might be acceptable. In our study, the addition of US to MG increased its sensitivity from 37.5 to 62.5%; therefore, MG + US might be considered another surveillance option if patients are unable to undergo AB-MRI due to cost or accessibility [34, 52]. However, there is still the possibility of delayed diagnosis of ipsilateral LTR caused by the ADM filler, especially in the case of LTR at the basal margin of the original tumor bed. Our study results can inform patient and clinician decision making regarding postoperative surveillance methods and be used to develop personalized screening guidelines and recommendations in these populations.

In our study, AB-MRI required less than 10 min for examination (mean, 8.5 min), which is comparable to the MG examination time. AB-MRI showed the highest sensitivity for the detection of breast cancer irrespective of breast density. However, there is no radiation exposure or breast compression during MG examination and no reproducibility issue for US examination. If AB-MRI is reimbursable for patients with a personal history of breast cancer, it can be a good screening test with a short examination time, low cost, low harm, and wide availability. However, gadolinium deposition within the basal ganglia by intravenous contrast injection is still a concern with frequent, repeated AB-MRI examinations.

Our study had several limitations. First, it was limited by its retrospective design with small populations. The CDR of our study might be overestimated due to the selection bias of our retrospective study from a single medical center. Second, the follow-up period of this study was relatively short (12–38 months), which could have affected the diagnostic performance of each modality. Third, we did not evaluate the appropriate interval and frequency of AB-MRI surveillance. In addition, studies on the cost-effectiveness and survival benefit of AB-MRI are needed for the wide application of this surveillance method. Our study indicates that a continued larger, prospective, multicenter study is needed to validate the benefit of AB-MRI surveillance in this population.

## Conclusions

In patients who underwent BCS with ADM volume replacement, AB-MRI showed an advantage for the detection of ipsilateral LTR despite postoperative changes. AB-MRI can be a useful postoperative surveillance tool for ipsilateral LTR considering its improved cancer detection and high diagnostic performance compared with MG and US.

## Data Availability

Please contact the corresponding author with requests for data.

## Abbreviations

BCS: Breast-conserving surgery
ADM: Acellular dermal matrix
LTR: Local tumor recurrence
MG: Mammography
US: Ultrasonography
MRI: Magnetic resonance imaging
CDR: Cancer detection rate
AB-MRI: Abbreviated MRI
BI-RADS: Breast Imaging Reporting and Data System
PPV: Positive predictive value
AUC: Area under the curve
ROC: Receiver operating characteristic
